# Promoting Healthy Decision-Making via Natural Environment Exposure: Initial Evidence and Future Directions

**DOI:** 10.3389/fpsyg.2020.01682

**Published:** 2020-07-14

**Authors:** Meredith S. Berry, Meredith A. Repke, Alexander L. Metcalf, Kerry E. Jordan

**Affiliations:** ^1^Human Behavioral Pharmacology and Decision-Making Laboratory, Department of Health Education and Behavior, University of Florida, Gainesville, FL, United States; ^2^Department of Psychology, University of Florida, Gainesville, FL, United States; ^3^Department of Psychology, University of Montana, Missoula, MT, United States; ^4^Department of Society and Conservation, University of Montana, Missoula, MT, United States; ^5^Department of Psychology, Utah State University, Logan, UT, United States

**Keywords:** environment, delay discounting, impulsivity, addiction, decision-making, nature, conservation, sustainability

## Abstract

Research within psychology and other disciplines has shown that exposure to natural environments holds extensive physiological and psychological benefits. Adding to the health and cognitive benefits of natural environments, evidence suggests that exposure to nature also promotes healthy human decision-making. Unhealthy decision-making (e.g., smoking, non-medical prescription opioid misuse) and disorders associated with lack of impulse control [e.g., tobacco use, opioid use disorder (OUD)], contribute to millions of preventable deaths annually (i.e., 6 million people die each year of tobacco-related illness worldwide, deaths from opioids from 2002 to 2017 have more than quadrupled in the United States alone). Impulsive and unhealthy decision-making also contributes to many pressing environmental issues such as climate change. We recently demonstrated a causal link between visual exposure to nature (e.g., forests) and improved self-control (i.e., decreased impulsivity) in a laboratory setting, as well as the extent to which nearby nature and green space exposure improves self-control and health decisions in daily life outside of the experimental laboratory. Determining the benefits of nearby nature for self-controlled decision-making holds theoretical and applied implications for the design of our surrounding environments. In this article, we synergize the overarching results of recent research endeavors in three domains including the effects of nature exposure on (1) general health-related decision-making, (2) health and decision-making relevant for application to addiction related processes (e.g., OUD), and (3) environmentally relevant decision-making. We also discuss key future directions and conclusions.

## Introduction

For decades environmental psychologists have extensively documented the multiple benefits to humans resulting from nature exposure. Although additional replication and extensions are needed, these benefits include a plethora of physiological and psychological improvements to human health including reduced recovery time following surgery, health improvements in patients with cancer (e.g., increased expression of anti-cancer antibodies), reduced hypertension, reduced stress, and increased happiness (Ulrich, 1984; Li et al., 2008; Mao et al., 2012; Thompson et al., 2012; White et al., 2013; see Twohig-Bennett and Jones, 2018 for a review and meta-analysis of health outcomes and greenspace exposure; see Houlden et al., 2018, for a review of the relationship between greenspace and mental wellbeing in adults). There is also recent evidence that suggests nature exposure could be a beneficial adjunctive treatment option in addition to traditional pharmacotherapy for individuals who suffer from disorders associated with lack of impulse control (e.g., addiction-related disorders, evidence from various fields reviewed in detail below). Despite these well-documented benefits of nature exposure to human health, we as humans continue to degrade our natural environments. The most critical environmental and public health crises that contribute to degradation of natural spaces (e.g., species extinction, forest degradation, accelerated climate change resulting from anthropogenic influence, millions of premature deaths annually resulting from emissions/poor air quality) are a direct result of human decision-making and behavior (Chivian and Bernstein, 2008).

For example, despite climate scientists’ account of current emissions as “dangerous to extremely dangerous” (Anderson and Bows, 2011), anthropogenic influenced global carbon emissions have surpassed the worst scenarios predicted by the Intergovernmental Panel on Climate Change (Boden and Blasing, 2010). Relatedly – poor air quality resulting from emissions is one of the leading causes of premature death worldwide – with nearly seven million mortalities occurring globally each year (World Health Organization [WHO], 2014, 2015, 2017). The direct contribution of human behavior (e.g., emissions from industries, factories, extensive private car use, air travel) in driving negative environmental outcomes (e.g., poor air quality, climate change) and resulting detrimental human health consequences (e.g., premature death) are well-documented and highly publicized.

In this manuscript we focus on the effects of nature exposure on decision-making processes relevant for human and environmental health. We will briefly synthesize findings and discuss future directions across key domains (1) at the intersection of nature exposure and human health related decision-making, (2) the potential for novel extensions combining health benefits and decision-making benefits of nature exposure to addiction research, and (3) the effects of nature exposure on environmentally relevant decision-making. The present manuscript is not an exhaustive literature review, but rather designed to briefly emphasize key discussion points and identify promising future directions related to nature exposure.

## Nature Exposure, Health, and Health Related Decision-Making

Our work has primarily focused on determining mechanisms that influence decision-making to result in healthier decisions for both humans and ecosystems. For example, decades of research have demonstrated that exposure to natural (e.g., forests, lakes) as opposed to built (e.g., cities, buildings) environments reduces stress (Ulrich et al., 1991; Thompson et al., 2012), enhances attention (Faber Taylor et al., 2001; Berto, 2005), and improves mood (Bowler et al., 2010). Beyond these psychological benefits, biodiversity is also crucial to our physical health for medicines, medical research, combating infectious diseases, and food production. Adding to the research on health and cognitive benefits of natural environments, this team’s research is among the first to show that not only cognition, but also *behavior, choice and delay discounting* are influenced differently as a function of natural versus built environmental exposure. Delay discounting refers to the decrease in value of an outcome with delay to receiving that outcome (Mazur, 1987). A delay discounting task evaluates choices between smaller sooner and larger later outcomes across a range of delays (e.g., $50 now or $100 in 5 years). A consistent pattern of choice of the smaller sooner outcomes is thought to represent relative “impulsive” decision-making. Delay discounting is one behavioral measure of “impulsivity.” Impulsivity has a number of different meanings (e.g., inability to delay gratification) and can be measured in different ways. High rates of delay discounting (i.e., “impulsive” decision-making) are associated with a host of maladaptive behaviors including cigarette smoking, opioid abuse, and gambling (see Odum et al., 2000; Dixon et al., 2003; Kirby and Petry, 2004; Mitchell, 2004a, b). Delay discounting, therefore, may represent a target for intervention for health-relevant behavioral processes. At present delay discounting is thought to be one of the most valuable decision-making predictors of human behavior both within the laboratory and real-world decision-making contexts (Chabris et al., 2008).

**FIGURE 1:**
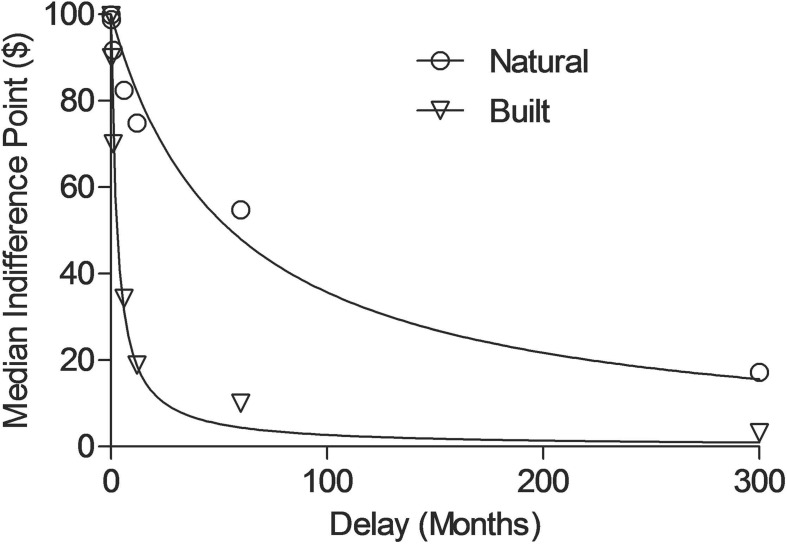
In this study, participants viewed photographs of either natural or built scenes on the computer screen prior to engaging in the delay discounting task and time perception task (see Berry et al., 2015 for additional details). The data points represent median indifference points (i.e., the subjective value) as a function of delay (months) for natural (circles) and built (triangles) conditions. Lines show the best fit of the non-linear regression equation to the median indifference points (see Berry et al., 2015 for additional details). The “steeper” curve shows more impulsive decision-making (built) and the shallower curve shows more “self-controlled” decision-making in the delay discounting task.

Some evidence shows choices in other delay of gratification tasks are more “self-controlled” (i.e., less impulsive) with exposure to nature as opposed to built environments. Faber Taylor et al. (2002) demonstrated that among children living in the inner city, the more natural a girl’s view from home was, the more “self-controlled” she was on a modified version of the classic marshmallow task (this same relation was not true for boys). van der Wal et al. (2013) also found that visual exposure to photographs of natural scenes on a computer screen resulted in less impulsive decision-making in a delay discounting task than photographs of built scenes. In a follow-up experiment, similar results were obtained when participants walked through either natural landscape environments or built landscape environments and then chose between receiving money now or in the future. Our lab has also shown that individuals visually exposed to natural environments exhibit more self-controlled decisions, while individuals exposed to built environments demonstrate more impulsive decisions in a monetary delay-discounting task (Berry et al., 2014; see Figure 1; Berry et al., 2015), and this effect may be related to expanded time and space perception (Berry et al., 2015; Repke et al., 2018).

Specifically, elongated time perception resulting from visual exposure to natural environments, could be a key mechanism underlying increased self-control with exposure to nature (Berry et al., 2015). It is possible that an expanded time perception window may facilitate bridging the gap between current decisions and future consequences of those decisions. The extent to which nearby nature and green space exposure improves self-control and health decisions in daily life outside of the experimental laboratory, however, has remained unexamined until recently, but holds applied implications for the design of our surrounding environments to promote healthy decision-making for individuals and surrounding ecosystems, as well as happiness and general well-being. We recently conducted two studies to test a new model linking the health benefits of nature exposure to reduced impulsivity in decision-making. We determined in a real world national United States sample, participants’ geospatial proximity to nature by quantifying the natural land cover surrounding participants’ home addresses using remotely sensed data. Measures of nature accessibility predicted reduced “impulsive” decision-making in a delay discounting task, and also showed significant indirect effects through impulsive decision-making on depression and anxiety measures and general health and well-being. We paired this study with a laboratory-based paradigm and found that visual exposure to nature expanded perceptions of space, and while the indirect effects of nature exposure through space perception on impulsive decision-making did not meet conventional standards of significance (*p* < 0.10), the pattern was consistent with hypotheses. This combination of ecologically valid and experimental methods offers promising support for an impulsivity-focused model explaining the nature–health relationship (Repke et al., 2018).

## Extending Current Applications of Nature Exposure and Health Decision-Making to Addiction-Related Research

Further extending and interweaving the above health-relevant decision-making models directly to addiction research, we recently proposed nature exposure as an adjunctive treatment option for opioid use disorder (OUD) in addition to traditional agonist pharmacotherapy treatment (e.g., methadone; Berry, under review). From 2002 to 2017 in the United States alone, deaths from opioid overdose have more than quadrupled (National Institutes on Drug Abuse, 2018). Relatedly, pain represents the leading cause of disability in the United States, affecting more Americans than diabetes, heart disease, and cancer combined (National Institutes of Health, 2019). The demand for opioid medication to effectively treat pain has contributed to the surging opioid crisis. More than 100,000 people begin opioid maintenance treatment (OMT) annually (Substance Abuse and Mental Health Services Administration [Samhsa] and Center for Behavioral Health Statistics and Quality, 2014), which is the standard of care. However (and paradoxically), OMT patients often experience or develop a heightened sensitivity to pain (hyperalgesia; Dunn et al., 2015), and have high rates of stress and affective and anxiety-related comorbidities (Hyman et al., 2007; Sinha, 2008; Gros et al., 2013). These conditions are interactive with other behavioral and environmental correlates of opioid and other substance use disorders including “impulsive” decision-making (e.g., harmful opioid use is associated with increased delay discounting), and a lack of alternative (i.e., substance free) and social (e.g., strong friendships and/or romantic relationships) reinforcement.

A promising, novel adjunct treatment option that could preserve the benefits of OMT and simultaneously improve pain management, decrease stress and anxiety, reduce behavioral correlates associated with OUD (e.g., “impulsivity” in delay discounting, or real world decision-making underscoring delay discounting processes in every day life, such as a choice between an immediate drug high over longer term healthy family relationships), as well as enhance alternative and substance free sources of reinforcement, may be exposure to nature and/or green space. Green light – similar to that in green spaces – has analgesic properties and reduces experimental pain in animal models (Ibrahim et al., 2017), an effect which appears to be mediated through the visual and opioid systems, and more. This effect has cross-species generality, showing that green light reduces pain intensity of migraines (Noseda et al., 2016), and nature scene murals placed at the bedside (as well as nature sounds in the background) during a flexible bronchoscopy procedure reduced self-reported pain compared to a control condition. This effect remained after controlling for age, gender, race, health status, and medication doses (Diette et al., 2003).

As discussed previously, research also shows exposure to nature (including green space) decreases anxiety, stress, and depression, which are common comorbid conditions among individuals with opioid use and other substance use disorders (Kushner et al., 1990, 2008; Koob and Schulkin, 2019). Access to green space and nature is also associated with reductions in craving of various substances (Martin et al., 2019). Given the strong relations between delay discounting and harmful opioid use that could serve as a potential therapeutic target, we reiterate that visual and actual exposure to natural environments also decreases “impulsive” decision-making in delay discounting tasks (e.g., van der Wal et al., 2013; Berry et al., 2015). Finally, individuals living farther away from recreational outlets (including parks and green space) and have less access to pleasant activities have higher rates of substance use, including prescription opioid use (Leventhal et al., 2015). Taken together, nature exposure could serve as a promising adjunctive treatment option for opioid abuse. However, little if any systematic research exists to inform this topic.

## Nature Exposure and Environmentally Relevant Decision-Making

In additional to personally relevant health decision-making, exposure to nature (either simulated or actual) may also promote environmentally relevant decision-making. Researchers have previously discussed the potential of nature exposure itself as a pro-environmental behavior trigger through various mechanisms (Annerstedt van den Bosch and Depledge, 2015). Zelenski et al. (2015) showed that people engage in future-oriented decisions that help to promote cooperation, conserve resources and behave more sustainably for themselves and team members in a public goods game after viewing videos of natural versus built environments These findings suggest that exposure to natural as opposed to built environments are not only beneficial for cognition, stress, and mood, but might also lead to healthier *behaviors* for individuals and ecosystems via improved global decision-making processes (i.e., decision-making across more than one domain, for example, money and health related decision-making, see Odum, 2011). These results have important implications for how we structure and design our environments (e.g., more green spaces in cities to promote future-oriented decision-making), as well as the impetus to preserve natural environments for human and ecological well-being.

Delay discounting, which has been proposed as a behavioral measure of sustainability (more sustainable associated with more “self-controlled” decision-making) and could serve as a global target for intervention. Few studies, however, have directly examined this concept in terms of environmentally relevant decision-making. Hardisty and Weber (2009) examined delay discounting of hypothetical financial, air quality, and health gains and losses scenarios across two delays. Within-subject analyses revealed that individuals who discounted gains steeply (“impulsively”) in one realm (e.g., monetary) also discounted gains steeply (“impulsively”) in other realms (e.g., air quality, health) and vice versa (see also Meyer, 2013; Johnson and Saunders, 2014; Kaplan et al., 2014; Richards and Green, 2015 for examples of environmental commodity discounting/support for long-term conservation goals). Extending this line of work Berry et al. (2017a, b) showed that mechanisms similar to those driving decisions for monetary outcomes might also be driving decisions about air quality (and possibly other ecological outcomes). This line of research lends support to targeting the same underlying mechanisms to facilitate reduction of delay discounting (“impulsivity”) on a global scale (see Odum, 2011). Therefore, reductions in monetary delay discounting as have been previously shown through various techniques (e.g., exposure to nature, van der Wal et al., 2013; Berry et al., 2015; future episodic thought, Peters and Büchel, 2010; for a review see Koffarnus et al., 2013) may also reduce delay discounting of air quality or other ecological commodities. Reducing delay discounting of environmental commodities could hold relevance for real-world environmentally relevant decision-making. We have further combined the above lines of research to determine if exposure to natural environments also decreases “impulsive” air quality choices. Evidence suggests that individuals respond in a more self-controlled way for ecological decisions related to air quality with exposure to natural as opposed to built environments (Berry et al., 2019). These results were associated with expanded space perception as previously shown (e.g., Repke et al., 2018). Although an initial foundation, this research still represents an area that has much opportunity for growth.

## Conclusion

More research is needed to further understand mechanistic drivers of behavior change with exposure to natural environments, and to test and expand these findings beyond laboratory settings. Exposure to natural environments provides a myriad of psychological benefits, and may also be useful as adjunctive treatments for addictive disorders (specifically OUD). Beyond personal health and wellness benefits, exposure to nature may also prove valuable for environmentally relevant decision-making.

As previously noted, there have been too few systematic experiments examining the potential of nature exposure as an adjunctive treatment for addiction (e.g., OUD) and disorders associated with lack of impulse control. This is also true of environmental decision-making in the context of nature exposure and actual behavior change outside of laboratory settings (i.e., not self-report data, although of course self-report data can be informative in different ways; please see Steg and Vlek, 2009 for review and commentary). Further, many of the studies reviewed here (and in the literature) focus on short-term effects. There is a need for longer-term and longitudinal studies to understand sustained benefits of nature exposure on psychological, health, decision-making and environmental outcomes. Experimental analysis of behavior might hold useful tools for identifying factors that increase or decrease discounting of environmental commodities – which could help shift individual and societal decision-making toward both long-term conservation and health-oriented behaviors. Of primary importance will be determining whether similar underlying processes drive environmental decision-making as monetary decision-making; and if these processes do share underlying mechanisms, reduction of impulsivity may be approached on a more global scale (see Odum, 2011 for discussion), holding implications for health, treatment of disorders associated with lack of impulse control such as OUD, and environmentally relevant decision-making.

## Ethics Statement

Ethical review and approval was not required for the study on human participants in accordance with the local legislation and institutional requirements. Written informed consent for participation was not required for this study in accordance with the national legislation and the institutional requirements.

## Author Contributions

MB synthesized the literature and wrote the original draft of the manuscript. All authors contributed to the conceptualization and review and editing of the manuscript draft.

## Conflict of Interest

The authors declare that the research was conducted in the absence of any commercial or financial relationships that could be construed as a potential conflict of interest.
